# Autoregulation of RNA Helicase Expression in Response to Temperature Stress in *Synechocystis* sp. PCC 6803

**DOI:** 10.1371/journal.pone.0048683

**Published:** 2012-10-31

**Authors:** Albert Remus R. Rosana, Danuta Chamot, George W. Owttrim

**Affiliations:** Department of Biological Sciences, University of Alberta, Edmonton, Alberta, Canada; University of British Columbia, Canada

## Abstract

RNA helicases are ubiquitous enzymes whose modification of RNA secondary structure is known to regulate RNA function. The pathways controlling RNA helicase expression, however, have not been well characterized. Expression of the cyanobacterial RNA helicase, *crhR*, is regulated in response to environmental signals that alter the redox poise of the electron transport chain, including light and temperature. Here we analyze *crhR* expression in response to alteration of abiotic conditions in wild type and a *crhR* mutant, providing evidence that CrhR autoregulates its own expression through a combination of transcriptional and post-transcriptional mechanisms. Temperature regulates *crhR* expression through alteration of both transcript and protein half-life which are significantly extended at low temperature (20°C). CrhR-dependent mechanisms regulate both the transient accumulation of *crhR* transcript at 20°C and stability of the CrhR protein at all temperatures. CrhR-independent mechanisms regulate temperature sensing and induction of *crhR* transcript accumulation at 20°C and the temperature regulation of *crhR* transcript stability, suggesting CrhR is not directly associated with *crhR* mRNA turnover. Many of the processes are CrhR- and temperature-dependent and occur in the absence of a correlation between *crhR* transcript and protein abundance. The data provide important insights into not only how RNA helicase gene expression is regulated but also the role that rearrangement of RNA secondary structure performs in the molecular response to temperature stress. We propose that the *crhR*-regulatory pathway exhibits characteristics similar to the heat shock response rather than a cold stress-specific mechanism.

## Introduction

The rearrangement of RNA secondary structure, required for numerous crucial cellular functions, is catalyzed by a variety of enzymes including RNA helicases. RNA helicases belong to a gene superfamily, SF2, members of which are encoded in essentially every organism from a variety of viruses to humans. SF2 is comprised of a number of protein families, with the DEAD-box proteins comprising the largest family [1]. RNA helicases function as molecular motors utilizing ATP hydrolysis to catalyze rearrangement of RNA and RNP structure with individual helicases performing specific functions potentially affecting all aspects of RNA metabolism [2]–[4]. Cellular pathways requiring RNA helicase activity include not only housekeeping functions such as translation initiation, ribosome biogenesis and RNA splicing and turnover but also developmental and stress pathways and small RNA metabolism [5]–[7]. Once RNA helicase expression is induced, the resulting RNA helicase activity has the potential to regulate expression of downstream genes required for the developmental or stress response.

Organisms utilize a variety of pathways to respond to temperature shift, the best characterized is heat shock while the mechanisms regulating the cold shock response are less defined. Cold stress induces expression of a limited number of genes, however, unlike heat stress, a sigma factor or two-component signal transduction system functioning as a global regulator of the response has not been identified [8]. This indicates that alternative mechanism(s) regulate gene expression in response to a temperature downshift in bacteria.

Frequently, RNA helicases are associated with the cold stress response in bacteria and higher organisms [9]–[15]. In prokaryotes, extensive analysis associates RNA helicase activity with ribosome biogenesis, RNA turnover and cold stress. Of the five *Escherichia coli* DEAD-box RNA helicases, *srmB* and *dbpA* are required for ribosome biogenesis, *rhlB* and *rhlE* with RNA degradation as a component of the degradosome and *csdA* is associated with both functions [11]–[12], [16]–[19]. Frequently, these functions are observed in response to cold stress, for example, at low temperature *csdA* and *rhlE* are degradosome-associated [16]–[17]. The association of RNA helicase expression and function with low temperature is also observed in photosynthetic Gram-negative cyanobacteria in which two DEAD-box RNA helicases have been studied, *crhC* and *crhR* [20]–[25]. *crhC* is expressed solely in response to low temperature [20]–[21] while *crhR* is regulated by the redox status of the electron transport chain, with expression increased by conditions that enhance reduction of the chain, for example low temperature or salt stress and dark-light transition [22], [26]. Despite the presence of numerous cold-induced RNA helicases in prokaryotes, the mechanism(s) by which their expression is regulated by temperature shift is not well defined.

**Figure 1:**
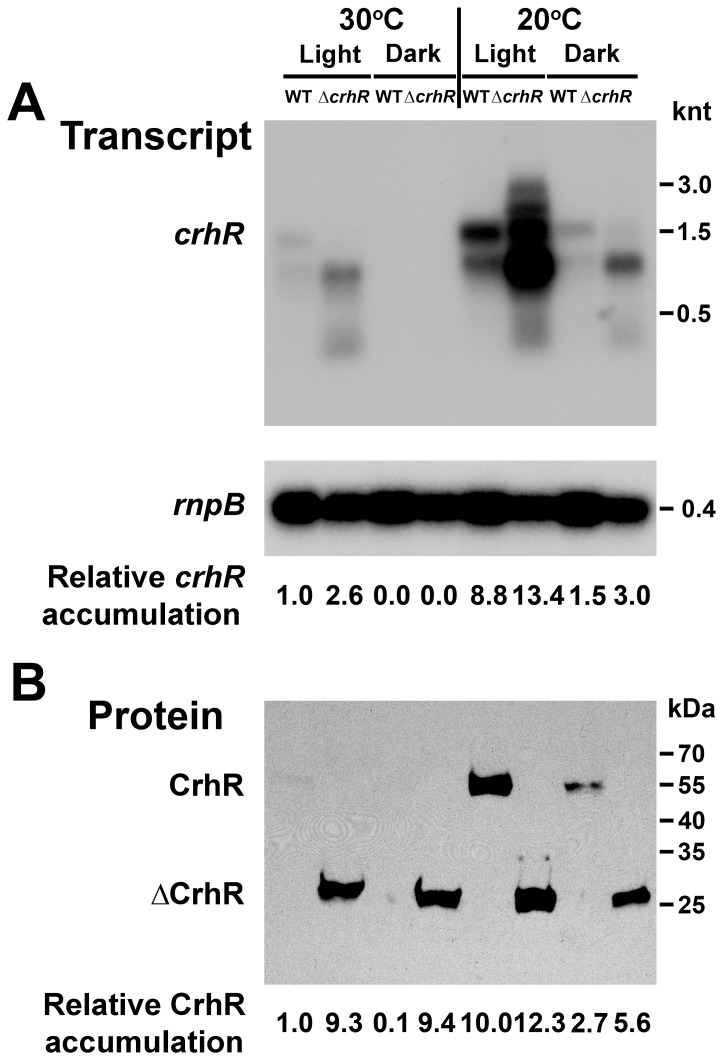
*crhR* expression in response to abiotic stress. (**A**) Northern analysis. Total RNA (5 µg) was probed with a 93 bp *Hinc*II-*Sac*II internal fragment of *crhR*. *Synechocystis* cells were grown at 30°C and stressed by transfer to the dark or 20°C for 1 h before harvesting at the indicated temperature. Shown below is the stripped blot probed with the *Synechocystis rnpB* as a control for RNA loading. Transcript abundance was quantified using Image J software Version 1.45 S (NIH, USA) [32], *crhR* transcript abundance was normalized using corrected *rnpB* levels with basal transcript abundance observed in illuminated wild type cells grown at 30°C set to 1.0 serving as a reference for the fold-change values shown (Methods S1). (**B**) Western analysis. Soluble protein (25 µg) isolated from the cells used for Northern analysis above was probed with anti-CrhR antibody. The anti-CrhR antibody detects a 55 kDa polypeptide in wild type cells and a ∼27 kDa truncated polypeptide in the Δ*crhR* mutant. Relative protein abundance is provided below each lane, in comparison with the abundance detected in illuminated wild type cells grown at 30°C set to 1.0.

At the physiological and morphological levels, *crhR* inactivation has profound effects on cellular metabolism at 30°C which are exacerbated at 20°C [27]. *crhR* mutants are cold sensitive with respect to both growth and photosynthetic activity, a phenotype resulting primarily from a defect in photosynthetic carbon fixation. These physiological effects are manifested morphologically by the *crhR* mutant progressively accumulating cellular damage at 20°C, including a reduction in the level and organization of carboxysomes and thylakoid membranes with the concomitant accumulation of membrane vesicles within the cells [27].

**Figure 2:**
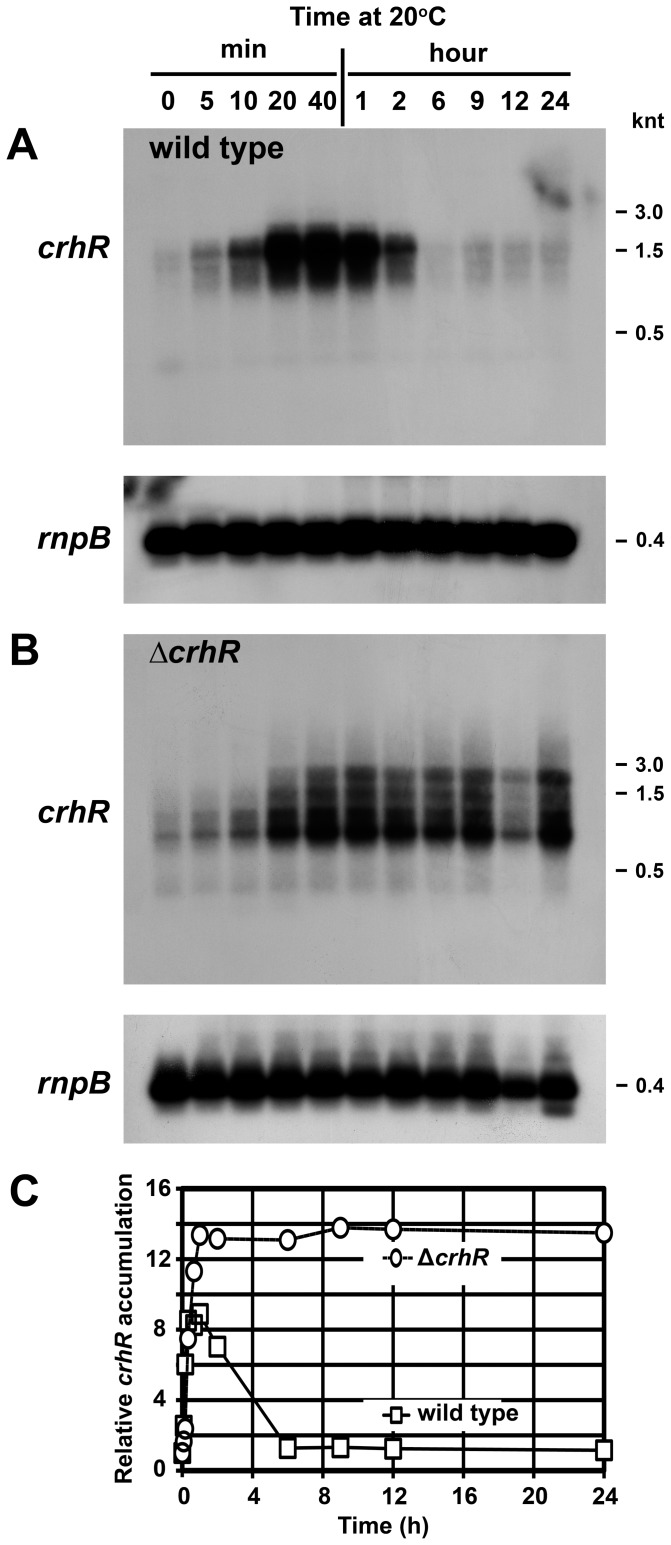
Time course of *crhR* transcript accumulation. Wild type (**A**) or Δ*crhR* mutant (**B**) *Synechocystis* were grown to mid-log phase at 30°C at which time the cultures were transferred to 20°C for the indicated times before harvesting. *crhR* transcript was detected in total RNA probed with a 93 bp *Hinc*II-*Sac*II internal fragment of *crhR*. The blots were stripped and probed with the *Synechocystis rnpB* gene as a control for RNA loading. **C**) Quantification of *crhR* transcript levels. Transcript levels were quantified as described in Figure 1 and expressed as accumulation relative to *crhR* abundance observed in illuminated wild type cells grown at 30°C (set to 1.0) serving as a reference for the fold-change values shown (Method S1). Open circles, Δ*crhR*; open boxes, wild type.

Here, we comprehensively investigate the molecular regulation of *crhR* expression in response to abiotic stress. In particular, we identify that temperature regulation of transcript and protein stability contributes significantly to *crhR* expression involving a unique combination of CrhR-dependent and CrhR-independent pathways. The results provide evidence that CrhR RNA helicase activity is required for transcriptional and post-transcriptional mechanisms that result in autoregulated expression.

**Figure 3:**
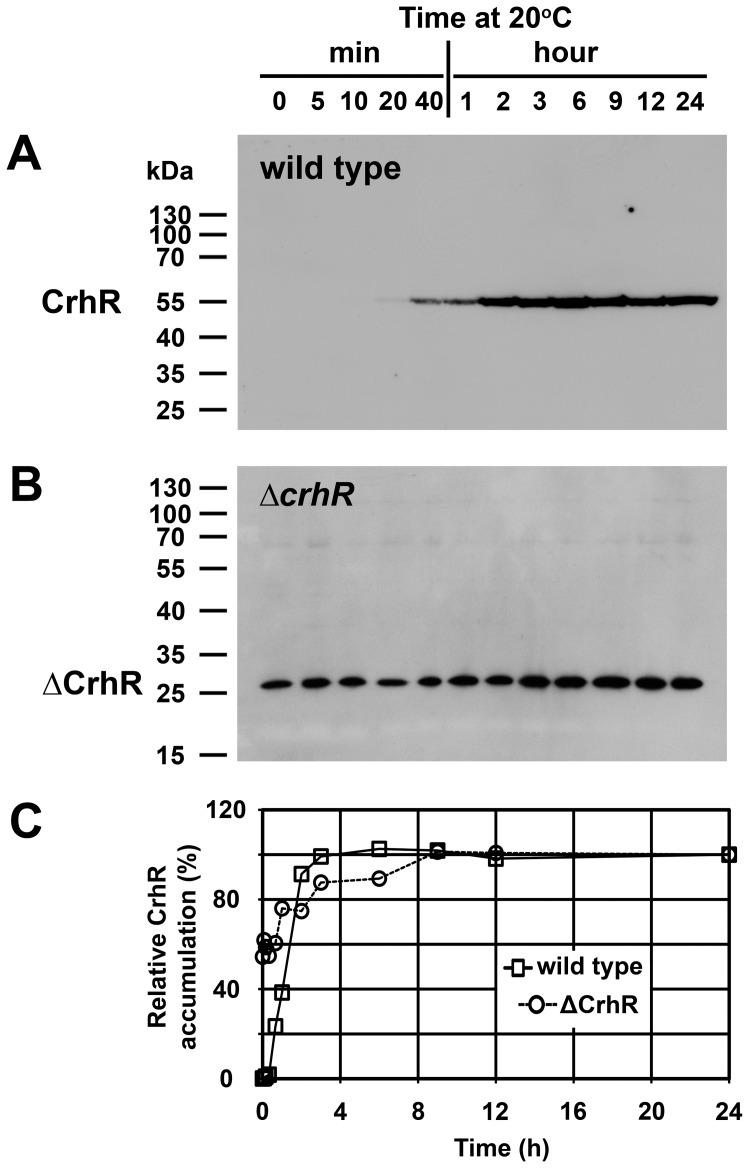
Time course of CrhR protein accumulation. CrhR in soluble proteins extracted from an aliquot of the cells used in Figure 2 was detected with an anti-CrhR antibody. (**A**) Wild type *Synechocystis*. (**B**) Δ*crhR* mutant. (**C**) Quantification of CrhR protein levels. Relative CrhR and ΔCrhR protein levels are indicated, normalized with respect to those present at 24 h, as described in Figure 1. Open circles, Δ*crhR*; open boxes, wild type.

## Materials and Methods

### Bacterial strains and growth conditions

Wild type *Synechocystis* sp. strain PCC 6803 and the partial deletion mutant, Δ*crhR* [27], were maintained on BG11-agar plates containing 10 mM Tricine pH 8.0 and 0.3% sodium thiosulphate at a light intensity of 50 μmol photons m^−2^ s^−1^. For liquid cultures, 50 mL BG-11 cultures were grown with shaking and used to inoculate 300 mL cultures that were aerated by continuous shaking and bubbling with humidified air [20]–[21], [28]. Media for *crhR* mutant growth was supplemented with spectinomycin and streptomycin, both at 50 µg/mL [27]. Temperature and dark stress were induced by transferring aliquots of cultures grown at 30°C to the indicated condition for 1 h. Cells for protein analysis were harvested at the stated growth temperature and cell pellets flash frozen in liquid nitrogen. For RNA extraction, cells and RNases were rapidly inactivated by addition of an equal volume of ice-cold ethanol-phenol buffer (ethanol−5% buffer saturated phenol) directly to the cell culture at the stated growth temperature. For temperature and half-life time course experiments, 400 mL cultures grown at 30°C were transferred to the indicated conditions and 50 mL aliquots removed at the indicated times for RNA and protein extraction. All experiments were repeated a minimum of two times with representative data shown.

**Figure 4:**
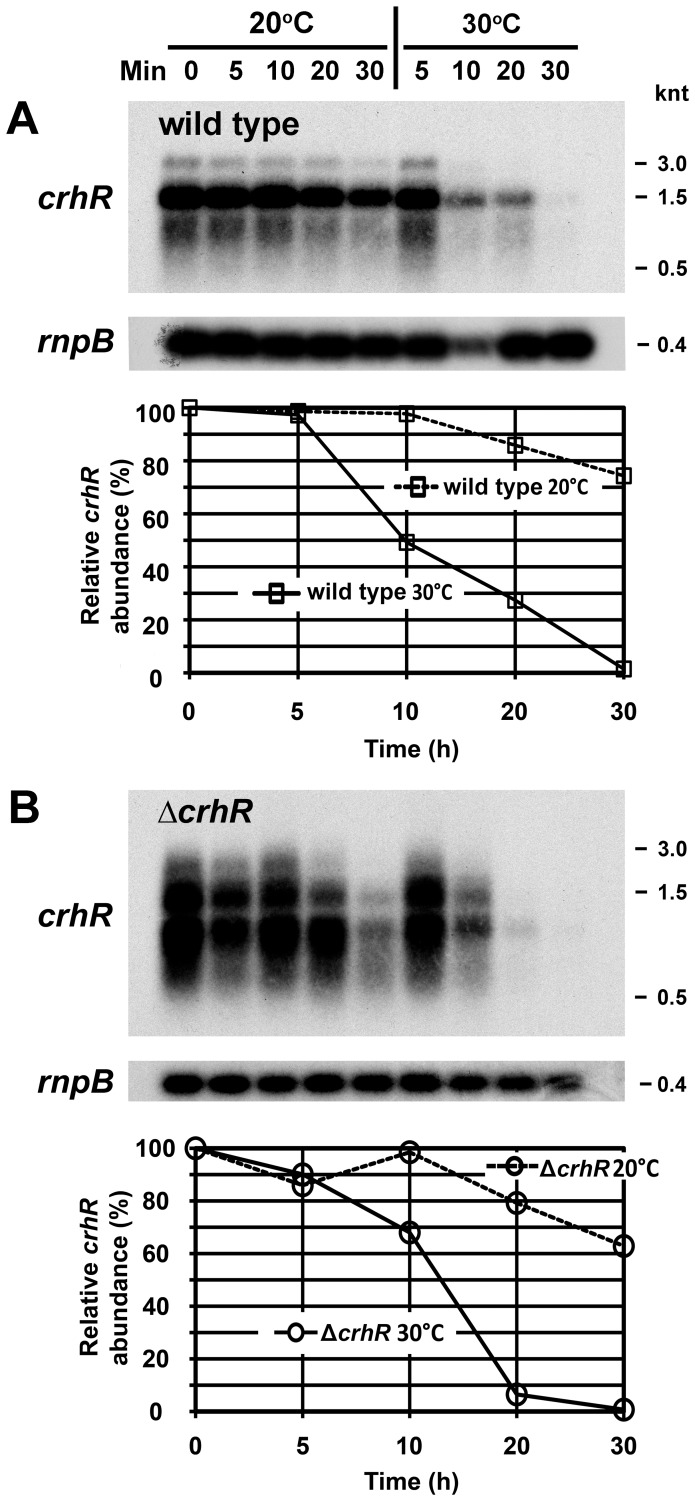
*crhR* transcript half-life. Wild type (**A**) and Δ*crhR* (**B**) *Synechocystis* were grown at 30°C to mid-log phase (0 min) at which time the cultures were cold stressed at 20°C for 1 h to induce maximal *crhR* transcript abundance. *de novo* RNA synthesis was subsequently inhibited by the addition of rifampicin (400 µg/ml) and one-half of the culture transferred back to 30°C. Aliquots for RNA extraction were harvested at the indicated times. *crhR* transcript was detected in total RNA probed with a 93 bp *Hinc*II-*Sac*II internal fragment of *crhR*. The blots were stripped and probed with the *Synechocystis rnpB* gene as a control for RNA loading. Quantification of relative transcript abundance (%) at each time point is provided below each lane, normalized for the level of *rnpB* detected in each lane as described in Methods S1. Open circles, Δ*crhR*; open boxes, wild type.

### RNA manipulation

RNA was extracted using glass bead lysis in the presence of phenol followed by extensive phenol-chloroform extraction and lithium chloride precipitation. Northern analysis, using a ^32^P-labelled riboprobe corresponding to a 93 bp *Hinc*II-*Sac*II internal fragment of the *crhR* ORF, was performed in formamide buffer at 65°C, as described previously [20]–[21], [28]. Transcript half-life was estimated in the presence of rifampicin (400 µg/mL), added to cultures immediately prior to transfer to the new temperature. Accumulation of the *Synechocystis rnpB* transcript was utilized as a control for RNA loading. The *rnpB* transcript abundance is extensively utilized for this purpose in cyanobacteria [29]–[31]. Transcript size was estimated using Fermentas RiboRuler™ RNA markers. Transcript levels were quantified using the Image J software Version 1.45 S (NIH, USA) [32].

**Figure 5:**
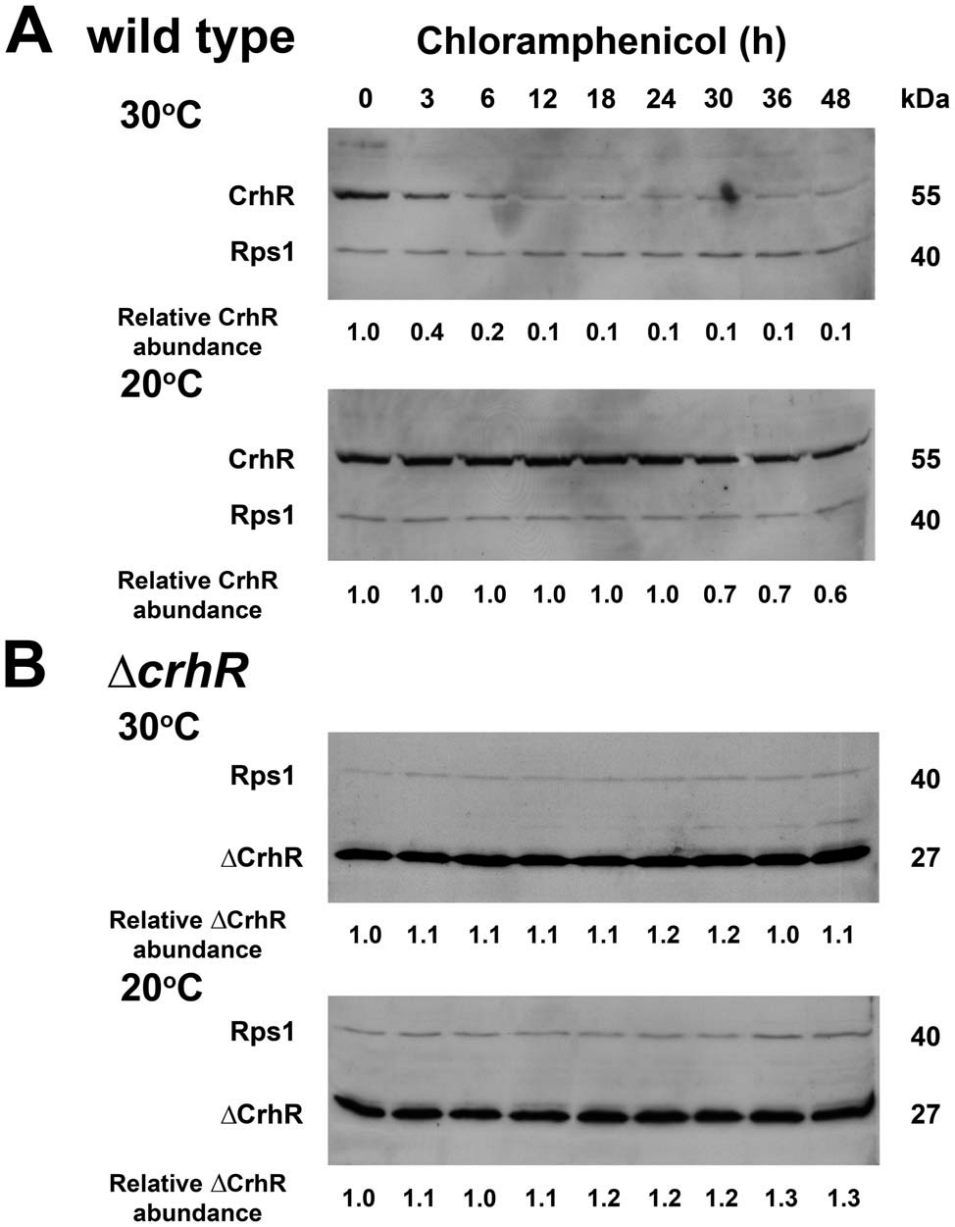
CrhR protein half-life. Wild type (**A**) and Δ*crhR* (**B**) *Synechocystis* were grown to mid-log phase at 30°C (0 min) at which time the cultures were transferred to 20°C for 2 h to achieve maximum CrhR and ΔCrhR abundance. Chloramphenicol (250 µg/ml) was added to inhibit *de novo* protein synthesis and one-half of each culture was transferred back to 30°C. Samples for soluble protein extraction were harvested at the indicated times. Blots were simultaneously probed with antibodies against CrhR and *E. coli* ribosomal protein S1 (Rps1), used as a control for protein loading. Quantification of relative CrhR and ΔCrhR protein abundance at each time point is provided, below each lane, normalized for the level of Rps1, as described in Methods S1.

### Protein manipulation

Protein extraction and immunoblot analysis were performed essentially as described previously [21], [28]. Soluble protein was extracted using glass bead lysis, 25 µg resolved by 10% SDA-PAGE, electro-transferred to Hybond ECL membrane and probed with the indicated antibody. Antibody complexes were detected on X-ray film using the Amersham ECL Western Blotting Detection kit. Polyclonal antiserum against *Synechocystis* CrhR or *E. coli* Rps1 was used at a dilution of 1∶5000. Rps1 levels were used as an internal control for protein loading. Protein half-life was determined in cells grown to mid-log phase at 30°C, transferred to 20°C for 2 h to achieve maximum accumulation of CrhR at which time chloramphenicol (250 µg/ml) was added to inhibit *de novo* protein synthesis and half of each culture transferred to 30°C. Protein concentration was quantified using the Bradford assay (Bio-Rad) with BSA as the standard. Protein levels were quantified using Image J software Version 1.45 S (NIH, USA) [32].

**Figure 6:**
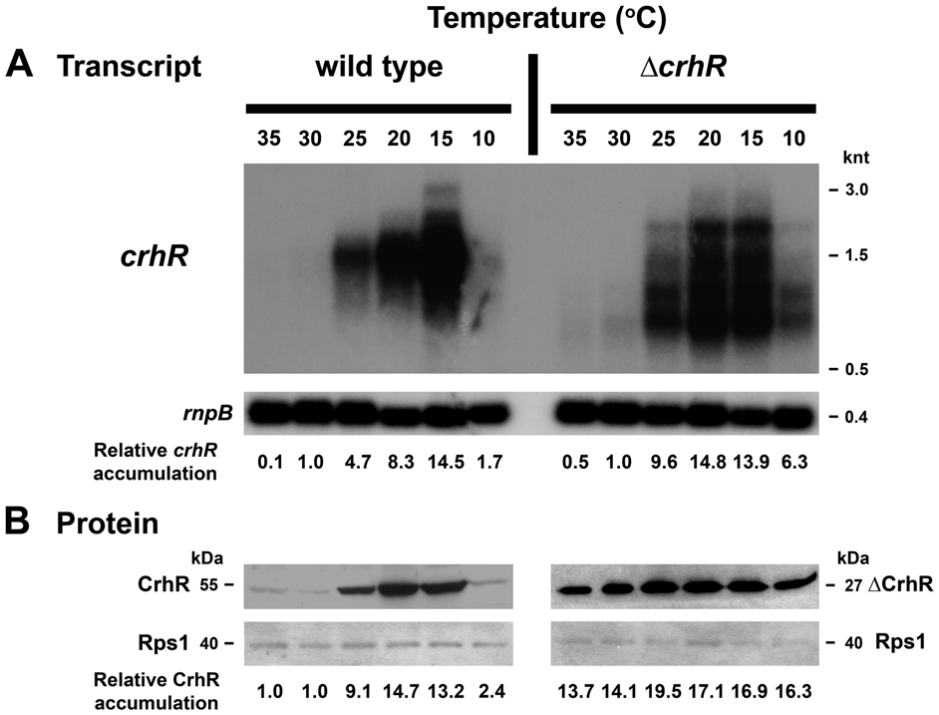
Temperature gradient of *crhR* expression. Wild type and Δ*crhR Synechocystis* were grown to mid-log phase at 30°C and divided into 6 aliquots, each of which was incubated at the indicated temperature for 1 h and samples were harvested for RNA and protein extraction. (**A**) Total RNA on a northern blot was probed with a 93 bp *Hinc*II-*Sac*II internal fragment of *crhR*, stripped and probed with the *Synechocystis rnpB* gene as a control for RNA loading. *crhR* transcript levels were quantified at each temperature as described in Methods S1 and the fold change in *crhR* accumulation compared to the abundance in illuminated, wild type cells grown at 30°C (set to 1.0) is provided below each lane. Ethidium bromide staining of the RNA present on the Northern blot indicates that essentially equal quantities of RNA were loaded in each lane (Figure S1). (**B**) Western blots were probed with anti-CrhR antiserum and subsequently probed with antibodies against *E. coli* Rps1 as a control for protein loading. Protein levels were quantified at each temperature and the fold change in CrhR or ΔCrhR compared to the abundance in illuminated, wild type cells grown at 30°C (set to 1.0) is provided below each lane, as described in Methods S1.

### ImageJ analysis

X-ray films were scanned using a UMAX PowerLook 2100XL scanner with the resolution set to 800 dpi. Scans were saved as tif files and imported into the ImageJ software package, available from http://imagej.nih.gov/ij/ [32]. The density of transcript and protein signals were plotted, corrected for background and integrated to give area values. Calculations normalizing for loading, correction of detected signals and determination of accumulation and abundance to quantify transcript and protein levels are described in Methods S1.

**Figure 7:**
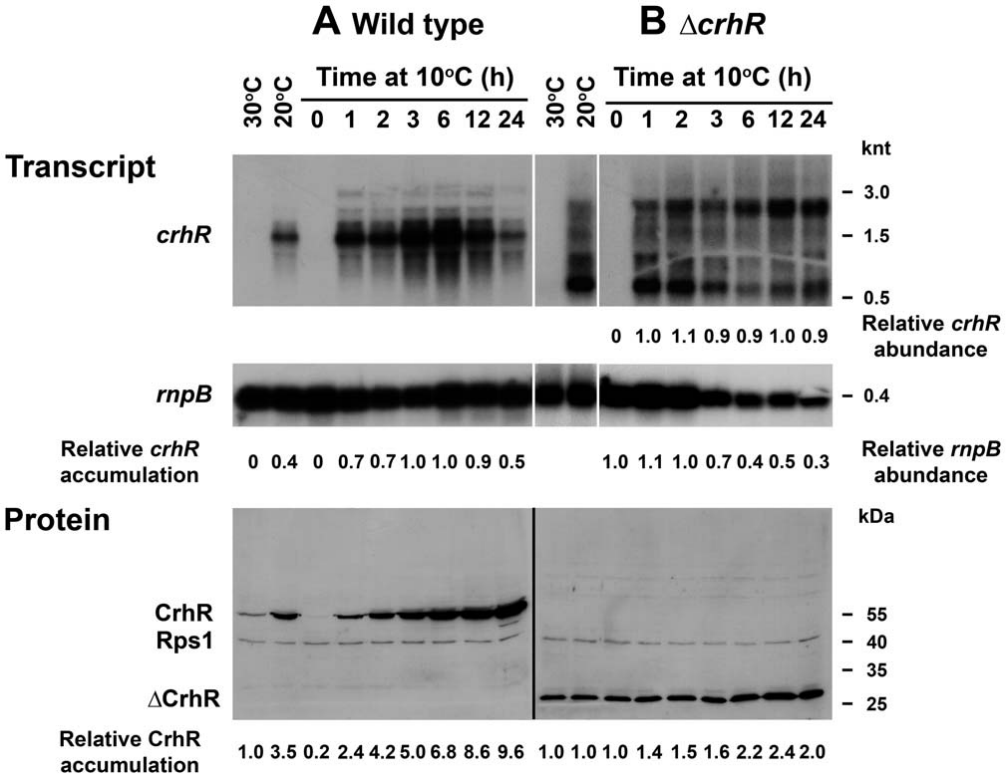
Time course of *crhR* expression at 10°C. Wild type (**A**) and Δ*crhR* (**B**) *Synechocystis* were grown to mid-log phase at 30°C and transferred to 10°C for the indicated times. Samples were harvested for RNA and soluble protein extraction at the indicated time points. Cells grown at 30°C and 20°C are included as references. (**A**) The relative *crhR* accumulation in comparison with the abundance in illuminated, wild type cells grown at 30°C corrected for *rnpB* levels (set to 1.0), is given below each lane for wild type cells. For Δ*crhR* cells, decreasing *rnpB* accumulation necessitated calculation of the relative abundance of *crhR* and *rnpB* independently. Transcript levels at 1 h of exposure to 10°C and 0 time were set to 1.0 for *crhR* and *rnpB*, respectively. Quantification of the 2.3 and 0.75 knt transcripts is shown in Table S1. (**B**) Western blots were simultaneously probed with antibodies against CrhR and *E. coli* Rps1 that served as a control for protein loading. The relative fold change in CrhR and ΔCrhR accumulation, corrected for Rps1 levels, as described in Methods S1, is given below each lane. Protein samples were resolved on separate gels, hence normalization was performed independently for the wild type and mutant, with abundance observed in wild type and mutant at 30°C set to 1.0.

## Results

### Induction of *crhR* expression in response to temperature and light-dark stress

We have extended our previous observations indicating that *crhR* expression is regulated in response to light-induced alteration of the redox poise of the electron transport chain [22] by investigating expression in response to temperature and light-dark transitions in wild type and *crhR* mutant *Synechocystis* (Fig. 1). In the *crhR* mutant, the ΔCrhR peptide is biochemically inactive, as it does not unwind dsRNA or anneal complementary ssRNAs (Chamot and Owttrim, unpublished). The fate of the truncated mRNA and protein products can therefore be investigated in the absence of biochemically active CrhR. In wild type cells grown at 30°C, a basal level of *crhR* transcript accumulation is observed, a level which decreases significantly in response to dark treatment for 1 h (Fig. 1A). *crhR* mutation altered this basal level as *crhR* transcript accumulation is enhanced 2.6-fold under standard growth conditions at 30°C in illuminated cells (Fig. 1A). However, regulation of transcript accumulation at 30°C was not completely lost in the Δ*crhR* mutant as transcript abundance, while elevated, was not increased to the levels observed under cold stress (Fig. 1A). While a predominant ∼1.5 knt *crhR* transcript was observed in wild type cells other stable, low abundance transcripts are also detected. *crhR* inactivation altered the transcript pattern, with four prominent stable transcripts of 2.3, 1.5, 1.3 and 0.75 knt accumulating in mutant cells. Although in wild type cells, the *crhR* probe detects multiple transcripts, they do not accumulate to the levels observed in the mutant. In response to temperature stress at 20°C for 1 h, *crhR* transcript levels increase and decrease substantially in the light and dark, respectively (Fig. 1A). However, similar responses to low temperature in both wild type and Δ*crhR* cells were observed, increasing significantly and, importantly, to approximately the same degree with respect to the basal levels observed at 30°C (Fig. 1A). Thus, *crhR* mutation altered the basal transcript abundance at 30°C but not the magnitude of the initial response to low temperature stress. Unexpectedly, accumulation of the *rnpB* transcript, coding for the functional RNA, RNase P, and used as a control for RNA loading, is marginally (8–10%) but consistently reduced in the Δ*crhR* mutant under all conditions tested (Fig. 1A). CrhR protein abundance corresponds with transcript accumulation in wild type cells with a basal level observed in the light at 30°C increasing significantly at 20°C and decreasing in response to dark treatment (Fig. 1B). In contrast, temperature regulation of protein accumulation was significantly altered in the Δ*crhR* mutant, in which the 27 kDa truncated CrhR polypeptide (ΔCrhR) was constitutively present at an elevated level, irrespective of temperature or light-dark stress (Fig. 1B). Interestingly, the enhanced abundance of the ΔCrhR peptide observed under all conditions was essentially identical to that observed in wild type cells at 20°C. Thus, in wild type and Δ*crhR* cells, CrhR protein levels do not correspond to transcript abundance and CrhR protein accumulates to a maximal level, irrespective of either transcript abundance or temperature.

**Figure 8:**
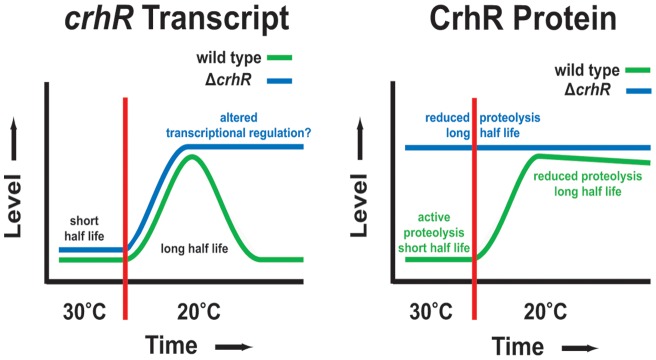
Schematic summary of *crhR* expression and regulation. *crhR* expression is controlled by a complex interaction between temperature-regulation of both transcript accumulation and protein degradation. ***crhR*** **transcript.** *crhR* transcript half-lives are equal and short in both wild type and Δ*crhR Synechocystis* cells which contributes to a basal level of transcript accumulation at 30°C. A temperature downshift to 20°C rapidly induces *crhR* transcript accumulation associated with enhanced half-life and with similar kinetics in both cell types, suggesting that CrhR is not required for temperature sensing or induction of its own transcript accumulation. At low temperature, *crhR* is transiently accumulated in wild type cells whereas conversely, transcript remains elevated at 20°C in the Δ*crhR* mutant, suggesting that CrhR activity is required for the transient expression. Although *crhR* half-life is influenced by temperature, being significantly longer at 20°C, it is identical in both cell types. This suggests that CrhR is not directly involved in degradation of its own transcript but functional CrhR is directly associated with repression of *crhR* transcript accumulation, most likely through another mechanism, possibly altered regulation of transcription. **CrhR protein.** CrhR protein levels correspond to transcript levels in wild type *Synechocystis*, accumulating to a basal level at 30°C and increasing significantly at 20°C. This is distinctly not the case in the Δ*crhR* mutant in which CrhR protein remains elevated and constant at both temperatures. CrhR protein accumulates to the level observed in wild type *Synechocystis* at 20°C, irrespective of transcript accumulation or temperature. Combined, these results suggest that the reduced level of CrhR at 30°C is caused by proteolytic degradation which is temperature- and CrhR-dependent as this process is inactive in the Δ*crhR* mutant. In addition, there appears to be a maximal level of CrhR that can accumulate in cells, irrespective of the transcript level, a process that is CrhR independent. This accumulation appears to be a default level as it was never observed to increase above the level detected in wild type cells at 20°C.

### Time course of *crhR* transcript accumulation

Alteration of the cellular response to temperature and light-dark stresses associated with CrhR inactivation prompted investigation of the kinetics of transcript accumulation. An essentially identical initial response in transcript accumulation is observed in both wild type and Δ*crhR* cells exposed to 20°C, transcript increasing in a linear fashion from a basal level at 30°C to a maximal level within 20 min (Fig. 2A and 2B). *crhR* transcript accumulation occurs transiently in wild type cells, decreasing to the basal level observed at 30°C within 6 h of exposure to cold stress (Fig. 2A and 2C). The transient expression occurring in wild type cells is not observed in the absence of functional CrhR, maximal *crhR* transcript levels accumulate after 20 min and remain consistently elevated for the duration of the experiment (Fig. 2B and 2C). Again, differential accumulation of four stable *crhR* transcripts was observed between wild type and mutant cells. This suggests that there is a defect in *crhR* transcript processing and/or degradation of the processed transcripts in the absence of CrhR activity (Fig. 2A and 2B).

### Time course of CrhR protein accumulation

CrhR protein accumulation in response to growth at 20°C also did not reflect the kinetics of *crhR* transcript accumulation in either cell type. In wild type cells, CrhR protein abundance corresponded with transcript levels during the early stages of the low temperature response but with delayed kinetics, reaching a maximum within 2 h of exposure. This correspondence did not hold at longer exposure times, with protein levels remaining elevated for the course of the experiment while transcript decreased significantly (compare Fig. 2A and Fig. 3A). As observed in Figure 1, temperature-regulated expression of the ΔCrhR polypeptide was absent in the Δ*crhR* mutant. ΔCrhR protein was expressed at relatively constant levels at all time points, with only a slight increase observed with extended low temperature exposure (Fig. 3B and 3C). Thus, again, *crhR* mutation resulted in a constant level of CrhR protein accumulation irrespective of transcript level or temperature.

### *crhR* transcript and CrhR protein half-life

The observed alterations in transcript and protein accumulation may reflect changes in macromolecular half-life. Indeed, *crhR* transcript abundance was regulated by temperature, but in a similar manner in both wild type and Δ*crhR* cells. Transcript stability was significantly enhanced at low temperature in wild type *Synechocystis* with half-lives of >30 and ∼10 min at 20°C and 30°C, respectively (Fig. 4A). Half-life was not altered by *crhR* mutation as similar values are observed in the Δ*crhR* cells, >30 min at 20°C and ∼13 min at 30°C (Fig. 4 B). These results suggest that the enhanced accumulation of *crhR* transcript or the lack of transient accumulation observed in Δ*crhR* cells in response to low temperature do not result from a defect in CrhR-dependent degradation of the *crhR* transcript.

Temperature regulation of protein half-life similarly controls CrhR accumulation in wild type cells (Fig. 5A). At 30°C, CrhR exhibited a relatively short half-life of <3 h which was increased significantly to >48 h at 20°C. In the Δ*crhR* mutant, ΔCrhR peptide levels remained elevated over the entire time course, not altering significantly in response to either chloramphenicol or temperature (Fig. 5B). Thus, while transcript half-life is not affected by *crhR* mutation, peptide half-life is significantly altered at both temperatures but more dramatically at 30°C, a response that is CrhR-dependent. The results indicate that *crhR* inactivation does not affect *crhR* transcript turnover but significantly affects CrhR protein turnover.

### Temperature gradient induction of *crhR* transcript and protein accumulation

It was of interest to determine if the temperature induction was a gradual or an all-or-none process. This was investigated by analyzing steady-state transcript and protein levels in *Synechocystis* exposed to temperatures ranging from 35°C to 10°C for 1 h (Fig. 6). At the transcript level, a basal level of *crhR* transcript accumulated at 35 and 30°C, which progressively increased to a maximum at 15°C (Fig. 6A). At 10°C, transcript accumulation was enhanced marginally, *crhR* transcript levels increasing 1.7-fold in comparison with the basal level detected at 30°C (Fig. 6A). CrhR protein expression in wild type cells mimicked transcript levels with the basal level of CrhR protein expression observed at 35 and 30°C increasing to a maximum at 20°C and subsequently decreasing to the basal level at 10°C (Fig. 6B). In the Δ*crhR* mutant, transcript abundance increases in response to decreasing temperature but reaches a maximum more rapidly and accumulates above basal levels at 10°C compared to wild type (Fig. 6A). As observed in Figure 3B, ΔCrhR protein levels in the *crhR* mutant remain essentially constant over the entire temperature gradient, increasing marginally in response to a 1 h exposure to temperatures ≤20°C (Fig. 6B).

### Time course of *crhR* transcript and protein accumulation at 10°C

The observation that *crhR* transcript levels were not enhanced in response to growth at 10°C indicates that a 5°C downshift in temperature dramatically affected cellular ability to respond to low temperature. This could result from either an inability to sense and/or respond at the transcriptional level or simply reflect a temperature-induced reduction in overall transcriptional activity. To further investigate this phenomenon, *crhR* transcript and protein levels were analyzed in response to prolonged exposure to 10°C. Overall, the time course of both *crhR* transcript and protein accumulation in wild type cells resembled that observed at 20°C (Fig. 2 and 3) however the kinetics were delayed at 10°C. In wild type cells, maximal transcript accumulation occurred within 3 h, plateauing and then decreasing up to 24 h (Fig. 7A). Conversely, CrhR protein accumulated progressively over the course of the experiment, even subsequent to *crhR* transcript decline (Fig. 7A), as also observed in Figures 2 and 3. In contrast, *crhR* transcript in the Δ*crhR* mutant was constitutively observed at all time points at 10°C (Fig. 7B, Table S1). The pattern of transcript accumulation differed from that observed at 20°C (Fig. 6A) with the 2.3 and 0.75 knt transcripts predominating at 10°C (Table S1). In addition, although the total accumulation of the four detected transcripts was not altered (Table S1), the relative accumulation of the two transcripts shifted in response to extended exposure to 10°C, with the 0.75 knt predominating at shorter and the 2.3 knt predominating at longer exposures, respectively (Fig. 7B, Table S1). This was not a result of alteration in total transcript, as quantification indicates that abundance of all four transcripts was constant over the experiment (Table S1). This is reflective of the results shown in Figures 2B and C, in both cases *crhR* transcript accumulation in the mutant is maximal within 1 h of exposure to 20°C and remains constant thereafter. These results imply that there is a defect in *crhR* transcript processing in the absence of CrhR RNA helicase activity. Again, ΔCrhR peptide levels remain relatively constant under all conditions tested, with only a marginal increase observed after prolonged exposure to 10°C, irrespective of RNA abundance (Fig. 7B). Similar to the results shown in Fig. 1A were *rnpB* transcript levels are consistently reduced under all conditions at 20°C, *rnpB* levels decreased progressively in response to extended growth at 10°C in the Δ*crhR* mutant (Fig. 7B). The observed decrease in *rnpB* accumulation would normally be an indication of unequal RNA loading, however analysis of total RNA in each lane, visualized by ethidium bromide staining, indicated that RNA loading was approximately equal in each lane (Fig. S1).

*crhR* expression was also evaluated in response to exposure of a single culture to a 5°C decrease in growth temperature for one hour, progressively from 35 to 10°C. In this scenario, a significant increase in *crhR* transcript level was detected in wild type cells in response to the temperature decrease from 15 to 10°C. This implies that wild type cells possess the capacity to respond to 10°C if they are cold adapted for one hour (data not shown). In agreement, CrhR protein levels also increase at 10°C in response to this temperature regime. As expected from experiments presented above, the level of the ΔCrhR polypeptide detected in mutant cells remained relatively constant, increasing slightly at temperatures below 20°C. Again, *rnpB* levels were observed to decrease at 10°C in the single culture exposed to the progressive 5°C decreases in temperature for 1 h intervals (data not shown).

## Discussion

The lack of a common global regulator of cold stress indicates that a variety of transcriptional and post-transcriptional mechanisms control gene expression in response to a temperature downshift [8]. The data presented here provide evidence that expression of the cyanobacterial RNA helicase, *crhR*, is controlled by a complex network of regulatory checkpoints involving both CrhR-dependent and CrhR-independent pathways, as outlined in Figure 8. Determination if CrhR is directly or indirectly involved in the autoregulation process and the mechanism by which the autoregulation functions are crucial questions. RNA helicases have well defined direct roles in translation initiation, RNA turnover and ribosome biogenesis in prokaryotic and eukaryotic systems [4], [19]. An indirect role for CrhR in autoregulatory mechanisms may be associated with one of these cellular pathways. For example, transient expression and proteolysis may originate indirectly from CrhR-regulated translation of transcripts whose protein products are required for these processes. An indirect role for CrhR in the observed regulation involving small RNA metabolism, as observed for RNA helicases in abiotic stress responses in eukaryotic systems [6] or on transcription cannot be ruled out. These may not reflect an indirect effect as p68 and p72 RNA helicases that catalyze similar biochemical reactions as CrhR [25], function as transcriptional co-regulators [33].

Temperature plays a profound role in *crhR* expression, enhancing both *crhR* transcript and protein half-life, although CrhR performs divergent roles in both mechanisms. CrhR-independent pathways are associated with temperature sensing, signal transduction and subsequent molecular response at the initial stages of cellular response to low temperature. The CrhR-independence of the initial response generating *crhR* transcript accumulation is related to the enhanced reduction of the electron transport chain at low temperature that is required for *crhR* transcription [22]. A major aspect regulating *crhR* transcript abundance is controlled by temperature alteration of *crhR* transcript half-life, unstable at 30°C and significantly stabilized at 20°C, as observed for other cold-regulated bacterial transcripts [21], [34]–[35]. The level of *crhR* transcript accumulation at different temperatures can potentially be explained by the temperature alteration of transcript stability, although we cannot rule out effects of CrhR regulation of transcription. The pathway conferring transcript stability is CrhR-independent, as *crhR* transcript half-life is not altered by *crhR* mutation. This observation suggests that CrhR is not directly involved in the RNA degradation pathway responsible for turnover of its own transcript. This is an important observation as RNA helicases frequently function in RNA turnover in prokaryotic systems, switching of RNA helicase composition in the RNA degradosome occurring in response to low temperature, resulting in formation of a cold-adapted degradation complex [36]–[38]. It is possible that CrhR is indirectly involved in degradation of its own transcript through regulation of translation initiation or another aspect of RNA metabolism associated with expression of the required RNase.

Transient accumulation of *crhR* transcript at 20°C and CrhR protein stability at all temperatures is CrhR-dependent. Transient transcript accumulation is a common characteristic of cold-induced genes in prokaryotic systems, although the mechanism is not well characterized [34], [39]–[40]. While transient accumulation in the absence of functional CrhR can also be interpreted as evidence that CrhR functions in the degradation of its own transcript, the lack of *crhR* transcript half-life alteration in the *crhR* mutant suggests that this is not the case. The kinetics of *crhR* transcript and protein accumulation more closely fit a scenario in which CrhR binding to the *crhR* transcript directs the CrhR-*crhR* complex to a CrhR-independent RNA degradation pathway. This proposal is similar to the direct mechanism by which proteolysis of the RNA chaperone CspC at high temperature regulates transient expression of heat shock mRNAs in *E. coli* [41], except that CrhR protein levels remain elevated continuously during temperature stress. Thus it appears that the CrhR-dependent transient accumulation of *crhR* transcript involves a direct association with RNA helicase activity, a role that does not involve RNA degradation.

CrhR peptide accumulation depends on a complex interplay between temperature regulation of both transcript and protein stability. The CrhR-dependent enhancement of CrhR peptide half-life at low temperature occurs in the absence of a correlation between protein and transcript levels. CrhR protein continues to accumulate in the absence of significant transcript accumulation at low temperature, similar to that observed for Rbp proteins in cyanobacteria [39]. This is in contrast to other cold-induced proteins where both transcript and protein transiently accumulate [34]. Changes in CrhR abundance could result from either altered translational efficiency or proteolysis. The increase in CrhR peptide abundance in the absence of functional CrhR at 30°C suggests that CrhR is not required for translation of its own transcript but is more likely indirectly involved to produce a component required for activity of the proteolytic degradation machinery. The mechanism does not appear to involve a general protein degradation pathway, as Rps1 levels are not significantly altered in these experiments. In addition, truncated CrhR polypeptide levels did not exceed those present in wild type cells at 20°C, suggesting that a CrhR-independent mechanism limits CrhR accumulation irrespective of transcript level or temperature. This implies that there is a maximum, default level to which CrhR can accumulate in *Synechocystis*, a process that is CrhR- and temperature-independent.

Proteolysis regulates the level of numerous proteins and regulatory circuits in bacterial systems, a prime example being the heat shock response [42]–[44]. In a natural environment, *Synechocystis* would normally experience temperatures below 25°C, conditions under which *crhR* expression would be constitutively induced. A temperature upshift to 30°C would enhance the proteolytic machinery that degrades CrhR. We therefore suggest that the results presented here resemble the regulation observed in response to heat stress rather than a cold stress specific phenomenon [44].

Regulation of prokaryotic gene expression is primarily thought to occur at the transcriptional level, with limited examples of control at the level of transcript or protein stability. The findings reported here, summarized in Figure 8, indicate that *crhR* expression is regulated in an unexpectedly complex manner, involving both CrhR-dependent and -independent pathways. CrhR-dependent pathways contribute to *crhR* expression via a novel combination of transcriptional and post-transcriptional mechanisms including autoregulation of the transient expression of *crhR* transcript at low temperature and CrhR protein accumulation at all temperatures. In contrast, aspects of temperature sensing, signal transduction, the initial increase in *crhR* accumulation and temperature-regulation of *crhR* transcript half-life involve CrhR-independent pathways. Frequently, these mechanisms regulate *crhR* expression in the absence of a correlation between protein and transcript levels. CrhR modulation of these divergent pathways coordinates cyanobacterial response to temperature fluctuation.

The data provide unique insights into the complexity of pathways regulating RNA helicase expression associated with bacterial response to temperature stress at the molecular level. Moreover, the research highlights the importance of RNA helicase remodeling of RNA secondary structure on downstream gene expression and the physiological implications for bacterial adaptation to temperature change.

## Supporting Information

Figure S1 **Ethidium bromide stained gel corresponding to the** ***crhR*** **induction time course at 10°C.** Total RNA (5 μg) extracted from wild type and *ΔcrhR Synechocystis* cells was separated on a 1.2% formaldehyde agarose gel at 100 V for 2.5 h. The gel was stained with ethidium bromide and imaged using a LKB 2011 Macrovue UV transilluminator equipped with a Kodak EDAS DC 290 camera and processed using Kodak 1D 3.6 imaging software. The ethidium fluorescence indicates that essentially equal amounts of RNA were loaded in each lane. Therefore, variations in *rnpB* transcript levels are not related to unequal RNA present in each lane.
(TIF)

Table S1 Quantification of *crhR* transcript hybridization detected in the Δ*crhR* mutant shown in Figure 7B.
(DOCX)

Methods S1 Quantification of RNA and protein abundance. The methodology and calculations used to quantify the relative transcript and protein levels using Image J [32], utilizing *rnpB* and Rps1 accumulation as controls for transcript and protein loading, respectively, are provided.
(DOCX)
