# Supplementary figures and images for: Autoregulation of RNA Helicase Expression in Response to Temperature Stress in Synechocystis sp. PCC 6803
Source: PLoS One. 2012 Oct 31;7(10):e48683. doi: 10.1371/journal.pone.0048683 (PMC3485376; doi:10.1371/journal.pone.0048683)

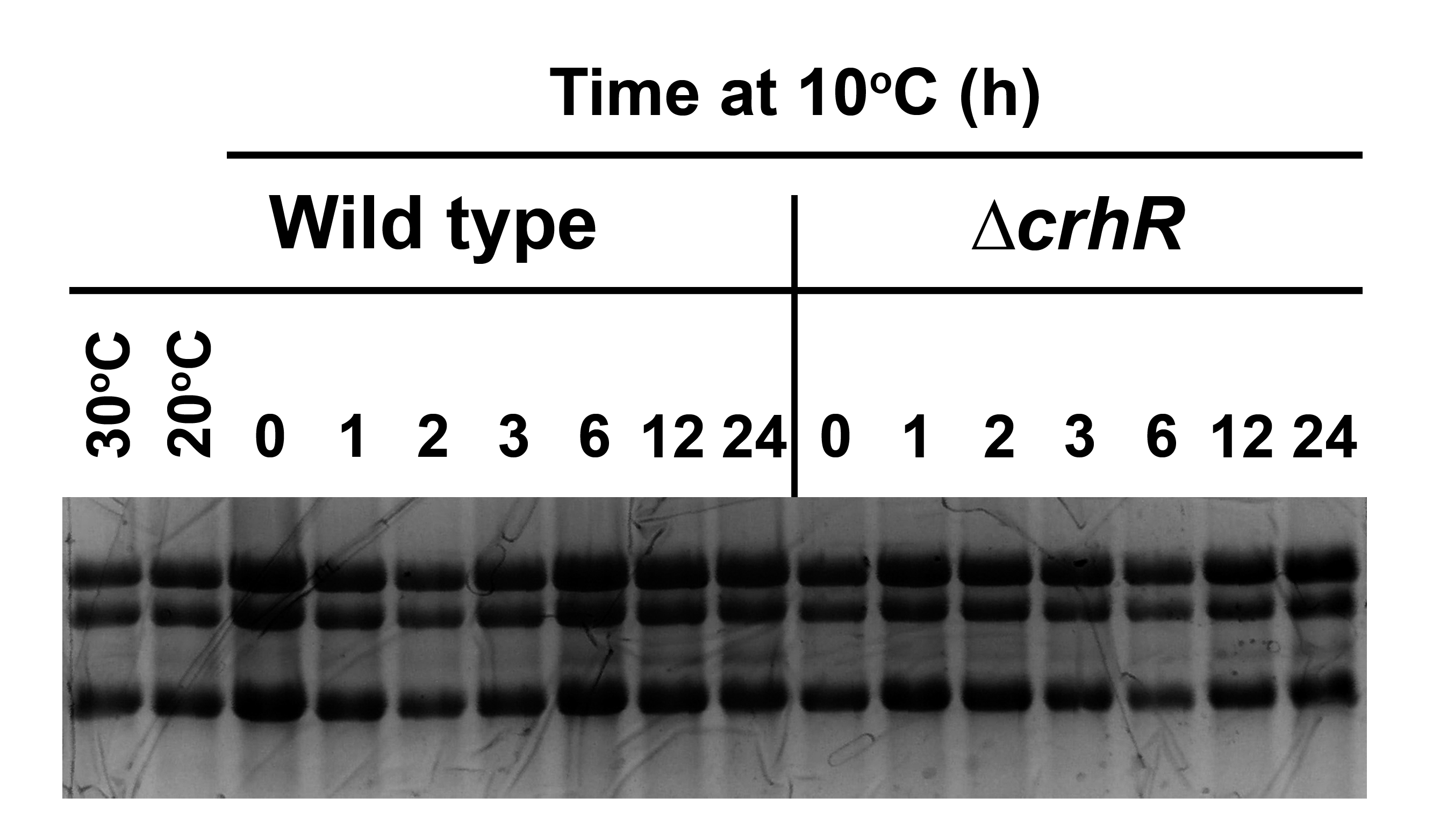

Supplement: Figure S1 — Ethidium bromide stained gel corresponding to the crhR induction time course at 10°C. Total RNA (5 μg) extracted from wild type and ΔcrhR Synechocystis cells was separated on a 1.2% formaldehyde agarose gel at 100 V for 2.5 h. The gel was stained with ethidium bromide and imaged using a LKB 2011 Macrovue UV transilluminator equipped with a Kodak EDAS DC 290 camera and processed using Kodak 1D 3.6 imaging software. The ethidium fluorescence indicates that essentially equal amounts of RNA were loaded in each lane. Therefore, variations in rnpB transcript levels are not related to unequal RNA present in each lane. (TIF) [file pone.0048683.s001.tif]
