# Supplementary material for: Autoregulation of RNA Helicase Expression in Response to Temperature Stress in Synechocystis sp. PCC 6803
Source: PLoS One. 2012 Oct 31;7(10):e48683. doi: 10.1371/journal.pone.0048683 (PMC3485376; doi:10.1371/journal.pone.0048683)
Supplement: Table S1 — Quantification of crhR transcript hybridization detected in the ΔcrhR mutant shown in Figure 7B. (DOCX) [file pone.0048683.s002.docx]

**Table S1 Quantitation of *crhR* transcript hybridization detected in the Δ*crhR* mutant shown in Figure 7B.**

| ***ΔcrhR*** | **Relative transcript level*** | | |
| --- | --- | --- | --- |
|  | **2300 nt*** | **750 nt*** | **Total *crhR***** |
| 10^o^C – 0 h | 0.00 | 0.00 | 0.00 |
| 10^o^C – 1 h | 1.00 | 1.00 | 1.00 |
| 10^o^C – 2 h | 2.16 | 0.92 | 1.10 |
| 10^o^C – 3 h | 1.09 | 0.65 | 0.90 |
| 10^o^C – 6 h | 2.34 | 0.30 | 0.90 |
| 10^o^C – 12 h | 3.57 | 0.56 | 1.00 |
| 10^o^C – 24 h | 3.23 | 0.65 | 0.90 |

*Individual transcript abundance was calculated by setting the level observed at 10^o^C for 1 h as 1.0.

**Total *crhR* abundance was quantitated using Image J analysis performed on the total area of hybridization in each lane.
