# Supplementary material for: Autoregulation of RNA Helicase Expression in Response to Temperature Stress in Synechocystis sp. PCC 6803
Source: PLoS One. 2012 Oct 31;7(10):e48683. doi: 10.1371/journal.pone.0048683 (PMC3485376; doi:10.1371/journal.pone.0048683)
Supplement: Methods S1 — Quantification of RNA and protein abundance. The methodology and calculations used to quantify the relative transcript and protein levels using Image J [32], utilizing rnpB and Rps1 accumulation as controls for transcript and protein loading, respectively, are provided. (DOCX) [file pone.0048683.s003.docx]

**Method S1**

**RNA QUANTITATION**

Transcript levels were quantified using the Image J software Version 1.45S (NIH, USA) available from http://imagej.nih.gov/ij/ [32] with *rnpB* as loading control [29, 30, 31]. Analysis was performed by normalizing *rnpB* in each lane with wild type 30^o^C *rnpB* levels set to 1.0. The normalized *rnpB* ratios were used to correct the abundance in the corresponding *crhR* lane. Corrected *crhR* values determined the fold change in each lane based on the basal accumulation observed in wild type cells grown at 30^o^C in the light which is set to 1.0 or maximum abundance (100%), as indicated. Corrected values were reported as either relative accumulation for time course analyses or relative abundance for half-life analysis.

Calculation of *crhR* transcript accumulation/abundance is provided below:

*Formula 1: Normalizing rnpB*

***rnpB* value E_X_**

**Normalized *rnpB* ratio E_x_**

***rnpB* value in WT at 30^o^C**

**=**

where;

*rnpB* value E_x_ = signal from experimental lane X

*rnpB* value of WT at 30^o^C = signal from wild type, 30^o^C grown cells, illuminated

*Formula 2: Correcting crhR transcript level*

***crhR* value E_x_**

**Normalized *crhR* level E_x_**

*from formula 1*

**Normalized *rnpB* ratio E_x_**

**=**

where;

*crhR* value E_x_ = *crhR* signal from experimental lane X

normalized *rnpB* ratio E_x_ = ratio of normalized *rnpB* in experimental lane X, value

obtained from *Formula 1*

*Formula 3: Relative crhR Accumulation or Abundance*

**Normalized *crhR* value E_x_**

**Normalized *crhR* levels in WT at 30^o^C**

**Relative *crhR* Accumulation E_x_**

*from formula 2*

**=**

where;

normalized *crhR* value E_x_ = corrected *crhR* signal from experimental lane X

normalized *crhR* levels at WT 30^o^C = corrected *crhR* signal from experimental lane WT

30^o^C, illuminated cell, value obtained from *Formula 2*

Note:

for Relative *crhR abundance* calculation, multiply the Relative *crhR* Accumulation E_x_ value by 100%

**PROTEIN QUANTITATION**

Protein levels were quantified using Image J software Version 1.45S (NIH, USA) available from http://imagej.nih.gov/ij/ [32] with Rps1 as loading control [28]. Normalization, correction and determination of relative CrhR protein levels were calculated essentially as described above for *crhR* transcript abundance, except that the abundance of ribosomal protein S1 (Rps1) was used as loading control. Rps1 levels were normalized for each lane using the abundance detected in wild type cells grown at 30^o^C in the light, set to 1.0 and used to normalize Rps1 levels in all lanes. The normalized Rps1 levels were used to correct for protein loading in each lane generating corrected CrhR abundances with the level observed in illuminated wild type cells grown at 30^o^C set to 1.0 or 100%. Corrected values were reported as either relative accumulation for time course analyses or relative abundance for half-life analysis.

Calculation of CrhR protein accumulation/abundance is provided below:

*Formula 1: Normalizing Rps1*

**Rps1 value E_X_**

**Rps1 value in WT at 30^o^C**

**Normalized Rps1 ratio E_x_**

**=**

where;

Rps1 value E_x_ = signal from experimental lane X

Rps1 value of WT at 30^o^C = signal from wild type, 30^o^C grown cells, illuminated

*Formula 2: Correcting CrhR protein level*

**CrhR value E_x_**

**Normalized Rps1 ratio E_x_**

**Normalized CrhR level E_x_**

*from formula 1*

**=**

where;

CrhR value E_x_ = CrhR signal from experimental lane X

normalized Rps1 ratio E_x_ = ratio of normalized Rps1 in experimental lane X, value

obtained from *Formula 1*

*Formula 3: Relative crhR Accumulation or Abundance*

**Normalized CrhR value E_x_**

**Relative CrhR Accumulation E_x_**

*from formula 2*

**=**

**Normalized CrhR levels at WT 30^o^C**

where;

normalized CrhR value E_x_ = corrected CrhR signal from experimental lane X

normalized CrhR levels at WT 30^o^C = corrected CrhR signal from experimental lane WT

30^o^C, illuminated cell, value obtained from *Formula 2*

Note:

for Relative CrhR Abundance calculation, multiply the Relative CrhR Accumulation E_x_ value by 100%
